# Performance evaluation of a scoria-compost biofilter treating xylene vapors

**DOI:** 10.1186/s40201-014-0140-4

**Published:** 2014-12-11

**Authors:** Mohammad Mehdi Amin, Amir Rahimi, Bijan Bina, Mohsen Heidari, Fazel Mohammadi Moghadam

**Affiliations:** Environment Research Center, Isfahan University of Medical Sciences (IUMS) and Department of Environmental Health Engineering, School of Health, IUMS, Isfahan, Iran; Student Research Center, School of Health, Isfahan University of Medical Sciences, Isfahan, Iran; Chemical Engineering Department, College of Engineering, University of Isfahan, Isfahan, Iran; Department of Environmental Health Engineering, Faculty of Health, Hormozgan University of Medical Sciences, Bandar Abbas, Iran; Department of Environmental Health Engineering, School of Health, Shahrekord University of Medical Sciences, Shahrekord, Iran

**Keywords:** Xylene, Biofilter, Scoria, Compost, Elimination capacity

## Abstract

The removal of xylene vapors was studied in a biofilter packed with a new hybrid (scoria/compost) packing material at various inlet loads (IL) and empty bed residence times (EBRT) of 90, 60, and 40s. The best performance was observed for EBRT of 90s, where a removal efficiency of 98% was obtained under steady state condition for inlet xylene concentration of 1.34 g m^−3^, while a maximum elimination capacity of 97.5 g m^−3^ h^−1^ was observed for IL of 199.5 g m^−3^ h^−1^. Carbon dioxide production rates and the microbial counts for xylene-degraders followed xylene elimination capacities. Overall look to the results of this study indicates that the scoria/compost mixture could be considered as a potential biofilter carrier, with low pressure drop (here <4 mm H_2_O), to treat air streams containing VOCs.

## Introduction

Xylene, together with benzene, toluene, and ethyl benzene, constitute the volatile organic compounds (VOCs) group BTEX. Xylene isomers namely *p*-xylene, *m*-xylene, and *o*-xylene are listed as hazardous and toxic atmospheric contaminants under Clean Air Act Amendments (CAAA) [1]. Chronic exposure to xylene is associated with adverse effects on the liver, kidneys and the central nervous system [2]. Xylene is a major constituent of gasoline and is used as a solvent in many production industries including printing, rubber, leather, painting and varnishing industries. In general, over 60% of the total emissions of xylene into the atmosphere originate from industrial facilities, especially petrochemical plants [1,3].

Among the air pollution control technologies, the biological treatment of VOCs provides an environmentally-friendly and low-cost alternative to other physicochemical treatment technologies [4-6]. The most widely used biological air treatment process is biofiltration [3]. The performance of biofilter significantly depends on the packing material properties [7]. Some of the desirable media properties include balanced chemical composition, relatively high water holding capacity, high microbial population density, and structural integrity [8,9]. These properties, except the later one, could be found in organic materials such as compost and peat, which allow the biofilter to have a quick startup and high elimination capacity. However, organic materials have a relatively low durability and tend to settle and compact, which in turn result in increased pressure drop and channeling [10]. On the other hand, the use of an inert material with rigid structure and large pores as filter bed allows a better gas distribution inside the reactor than organic carriers and minimizes the pressure drop build ups [11,12]. Consequently, using a mixture of organic and inert material inoculated with acclimated microbial consortium not only might improve the performance of biofilters, but also provides low pressure drops.

Many studies have been conducted on the biofiltration of xylene from waste air streams mainly in mixture with other VOCs. Most of them demonstrated that the removal of xylene isomers is always less efficient among the BTEX compounds [13-16]. In other hand, the biofiltration of xylene as the sole pollutant has been less considered. Saravanan and Rajamohan [1] studied the biofiltration of xylene vapors on a laboratory scale biofilter packed with press mud as filter material inoculated with activated sludge and reported a maximum elimination capacity of 67 g m^−3^ h^−1^ for inlet load of 103 g m^−3^ h^−1^ and EBRT of 42 s.

To the best of our knowledge, no attempt has been made on the removal of VOCs in biofilters packed with a mixture of scoria and compost. Scoria is a vesicular pyroclastic rock with basaltic composition, which is light in weight, porous, and cheap. It is abundant in many places worldwide [17]. In this study a mixture of scoria (pre-soaked in an adapted inoculum) and compost was used for the biofiltration of xylene. The aim of this study was to evaluate the performance of the biofilter under various operating conditions in terms of xylene removal efficiency and elimination capacity. The carbon dioxide evolution and microbial population distribution along the bed length were also monitored.

## Materials and methods

### Inoculum and packing media preparation

Scoria and compost were used as the biofilter media. The media characteristics are given in Table 1. Scoria, as the inert porous media, was used to ensure more homogeneous gas distribution across the filter bed, to maintain the structural integrity of the biofilter and to minimize the pressure drop. Activated sludge from a wastewater treatment plant located at Isfahan Petroleum Co. (Isfahan, Iran) was used as the inoculum source for the biofilter. Microorganisms in the activated sludge were acclimated to xylene in order to reduce the required adaptation time for the microorganisms in the biofilter. For acclimation, one liter of the activated sludge was placed in an aerated batch reactor and diluted with 3 L of nutrient solution. The used nutrient solution was composed of (g l^−1^ of tap water): 2.36 (NH_4_)_2_SO_4_, 2.09 KH_2_PO_4_, 1.91 NH_4_Cl, 0.27 Na_2_HPO_4_.7H_2_O, 0.5 NaCl, 0.03 CaCl_2_.2H_2_O, 0.2 MgSO_4_.7H_2_O, 0.03 ZnSO_4_.7H_2_O, 0.03 FeCl_3_.7H_2_O, 0.03 MnSO_4_. Xylene as sole carbon source was injected to the reactor at rates of 5–10 ml day^−1^. In order to avoid metabolic by-product accumulation and nutrient deficiency, one liter of the mixture was substituted with fresh nutrient solution on weekly basis. After two months, 5 liter of solution nutrient was added to the mixture and shaken vigorously, and then around 3 kg of dried scoria was soaked in the mixture. Before packing, the prepared scoria stones were mixed with compost in a 3:1 volume ratio.

**Table 1:** **Biofilter media characteristics**

**Characteristic**	**Fresh scoria**	**Compost**
Particle size (mm)	6-13	1-6
Bulk density (kg m^−3^)	444	325
Initial moisture (%)	<2	26.5
Water holding capacity (kg kg^−1^ dry weight)	43	125
pH (−)	7.9	8.5
Porosity (%)	70	58
C/N ratio (−)	-	18
Special surface area (m^2^ g^−1^)	0.282	-

### Experimental setup

**Figure 1:**
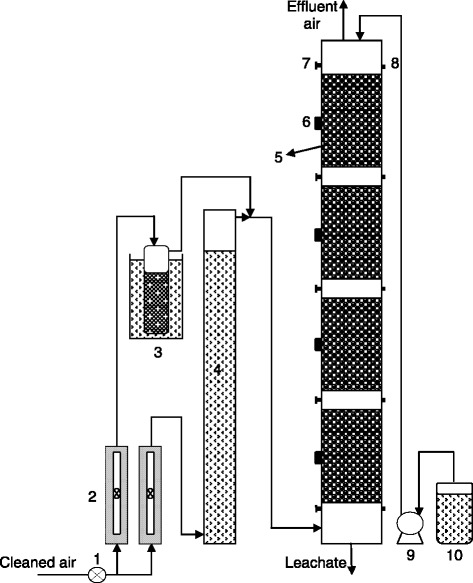
**Schematic of the biofiltration set up.** 1. Air pressure regulator, 2. Air rotameters, 3. Water bath and bubbler, 4. Humidifier, 5. Biofilter bed, 6. Bed sampling port, 7. CO_2_, temperature and pressure drop measurement port, 8. Gas sampling port for xylene measurement, 9. peristaltic pump, and 10. Nutrient solution container.

Experiments were carried out in a four-stage laboratory-scale biofilter made of stainless steel with an effective bed height of 80 cm and an internal diameter of 11.5 cm (Figure 1). Perforated stainless steel plates were located at the bottom of each section to support the packing material and to enhance the radial distribution of gas between the sections. A 10-cm space between adjacent sections allowed for gas sampling and redistribution of the contaminant streams. Gas sampling ports were installed at the inlet, outlet and at additional spaces between adjacent sections. Furthermore, a port was located at the middle of each section to recover bed particles for additional analyses. The filter bed was irrigated daily with 1 liter of the previously mentioned nutrient solution. The leachate was manually collected on a daily basis at the bottom section of the biofilter.

Compressed air was continuously passed through a granular activated carbon column to remove moisture, oil and particulate matter. The purified air was divided into two streams; the major one was passed through a water column in order to increase the relative humidity of inlet air and the minor one was bubbled through a glass bubbler containing xylene solution (Merck, Germany). The bubbler was held in a thermostatically-controlled water bath. Afterward the streams were mixed and fed to the bottom of the biofilter column in a counter-current flow mode. Xylene concentration was maintained at the desired value by adjusting the flow rate of air passed through the bubbler, temperature of water bath, and the level of pollutant in bubbler.

### Analytical methods

Gas phase xylene was measured by a gas chromatograph equipped with flame ionization detector (GC-FID) (Agilent GC, 7890A). The column used was an Agilent 19091S-433 capillary column, 30 m × 250 μm × 0.25 μm. High purity helium gas (99.995%) was the carrier gas and supplied at a flow rate of 1.11 ml min^−1^. The temperatures of column oven, injector and detector were 150, 230 and 250°C, respectively. The method detection limit (MDL) for xylene analysis was estimated as 11.1 μg l^−1^. The calibration curve was prepared by injecting known amounts of the xylene into a glass bottle sealed with Teflon septum according to the standard procedure [18]. Air samples of 100 μl were drawn from the bottle and various sampling ports along the biofilter column with a 1 mL gas tight syringe (Hamilton, USA), and directly injected into GC. A Guardian Plus Model D100 IR CO_2_ analyzer (Edinburgh Sensors Ltd., Munchen, Germany) (0–3000 ppm) was used to analyze carbon dioxide concentration along the biofilter column. Total organic compounds (TOC) in the leachate were measured using a TOC analyzer (Beckman 915A, USA). In order to break microbial flocs into small pieces and achieve a homogeneous TOC concentration, the leachate sample was surged by ultrasonic wave. The moisture content of packing material was determined as weight loss after drying at 105°C for 24 hr.

The temperatures at midlevel of each section and at gas sampling ports were measured by online K-type thermocouple and a digital thermometer, respectively. The pressure drop across the biofilter was measured using a water column manometer. Scanning electron micrographs of the scoria were carried out using a Vega/Tescan scanning electron microscope (SEM). For the sample taken at the end of experiment, the microorganisms were fixed with 3% glutaraldehyde aqueous solution overnight. The fixed sample was washed with phosphate buffer, dehydrated by placing in 30%, 50%, 70% and 100% ethyl alcohol, dried and then covered with a gold layer.

### Microbial cell counts

Almost one gram of the packing material was taken from each section of the biofilter and mixed with 9 mL sterile saline solution (0.9% w/v NaCl). The samples were then vortexed for 3 min and serially diluted up to 10^−10^ in sterile saline solution. Aliquots of 0.1 ml were spread over the surfaces of agar plates. The Nutrient Agar amended with Nistatin (to inhibit fungal growth) and the Potato Dextrose Agar (PDA) amended with chloramphenicol (to inhibit bacterial growth) were used for culturing bacteria and fungi, respectively. Furthermore, a sterile mineral medium containing 1.5% (w/v) agar was used for the enumeration of xylene-degraders. Xylene vapor as sole carbon source was supplied through placing plates in a sealed container. The cultured plates were incubated at 28–30°C for 5–7 days. The counts were reported as colony forming unit (CFU) g^−1^ of packing material (dry basis).

### Performance evaluation

Biofilter performance was evaluated in terms of inlet xylene load, IL (g m^−3^ h^−1^); elimination capacity, EC (g m^−3^ h^−1^); removal efficiency, RE (%); and carbon dioxide production rate, PCO_2_ (g m^−3^ h^−1^). These parameters are defined as:

1 $$ IL=\frac{Q{C}_{\mathrm{in}}}{V} $$

2 $$ EC=\frac{Q\left({C}_{\mathrm{in}}-{C}_{\mathrm{out}}\right)}{V} $$

3 $$ RE=\frac{C_{\mathrm{in}}-{C}_{\mathrm{out}}}{C_{\mathrm{in}}}\times 100 $$

4 $$ PC{O}_2=\frac{Q\left(C{O}_{2,\ \mathrm{out}}-C{O}_{2,\  in}\right)}{V} $$

where Q is the air flow rate (m^3^ min^−1^), V is the volume of packed bed (m^3^), C_in_ and C_out_ are the inlet and outlet xylene concentrations (g m^−3^), and CO_2,in_ and CO_2,out_ are the inlet and outlet carbon dioxide concentrations (g m^−3^), respectively.

## Results and discussion

### Overall performance of biofilter

**Figure 2:**
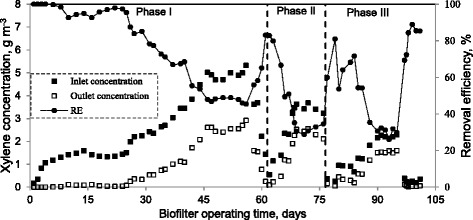
**The variation of the inlet and outlet xylene concentrations and removal efficiency of the biofilter as a function of time.**

The biofiltration of xylene vapors was carried out during 101 days at various operating conditions, which are listed in Table 2. In each phase, the inlet concentration of xylene was increased gradually to the high levels in order to envisage the maximum performance of the biofilter. The variation of inlet and outlet xylene concentrations and removal efficiency of the biofilter as a function of time are presented in Figure 2.

**Table 2:** **Biofilter operating conditions**

**Phase**	**I**	**II**	**III**
Days of operation	1-61	62-76	77-101
EBRT (s)	90	60	40
Inlet concentration range (g m^−3^)	0.17-5.32	0.54-3.68	0.14-2.27
Inlet loading rate range (g m^−3^ h^−1^)	6.9-212.8	32.3-220.9	12.6-204.6

**Figure 3:**
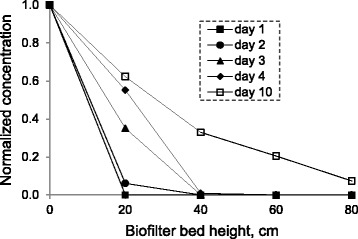
**Normalized xylene concentration profiles along the bed height of biofilter at initial days of operation.**

The system was started up with a gas flow rate of 5.5 l min^−1^, resulting in an EBRT of 90 s with an inlet xylene concentration of 0.17 g m^−3^. The removal efficiency of the biofilter was 100% for first day. This means that no acclimation time was needed for the microorganisms to remove xylene effectively. The variation of xylene concentration through the bed length shows that for initial days of operation, all the incoming xylene was removed through the first and second sections of the bed (Figure 3). Therefore, based on the above mentioned observations and to ensure the biofilm growth on all surfaces of the media through the bed length, the inlet concentration of xylene was increased in a rapid manner. By increasing the concentration up to 1.42 g m^−3^, the removal efficiency decreased to 92.6% at day 10. The complete removal of xylene in the initial days may be attributed to the presence of highly adapted microbial populations along with the adsorption of xylene on the packing medium of the biofilter. Inoculation of the biofilter media with adapted microbial aggregates minimizes the acclimation time of the biofilter [19]. As noted previously, scoria was inoculated with an adapted microbial culture before packing. This phenomenon increased the number of xylene-degraders up to 1 × 10^8^ CFU g^−1^ dry scoria, which was almost 380-fold higher than the initial cell concentration reported for an inoculated compost-based biofilter treating p-xylene vapors (i.e. 2.65 × 10^5^ CFU p-xylene degraders g^−1^ dry compost) [20].

During phase I, the inlet concentration was increased slowly in order to prevent shock loading of biofilter and to reach a maximum performance (EC_max_). From day 10 to 25, the inlet concentration was varied from 1.32 to 1.58 g m^−3^ and the removal efficiency was maintained over 92.6%. The removal efficiency decreased significantly to 53.6% as the xylene concentration increased to 4.47 g m^−3^ by day 44. From days 46 to 56, the inlet concentration was raised and maintained at highest levels during the whole experiment, i.e. 4.7 to 5.3 g m^−3^ with corresponding loads of 187.8 to 212.8 g m^−3^ h^−1^. In this period a pseudo steady state was achieved, since the xylene removal efficiencies varied from 45.3 to 47.9%. According to Rahul et al. [16] the pseudo steady state was presumed when the changes in the xylene removal efficiency were within 5% for three successive days.

The next two phases of operation were aimed at investigating the biofilter performance under lower EBRTs. In phase II, the EBRT was reduced to 60 s and the biofilter could provide over 80% removal of xylene for the inlet concentrations below 1.14 g m^−3^. The removal efficiency sharply declined to an average of 33.4 ± 4.1% during the days 68–76, when the inlet xylene concentration was increased to a range of 3.22 to 3.68 g m^−3^, with loading rates of 193.4-220.9 g m^−3^ h^−1^. The last phase of the experiment commenced on day 77; the EBRT was further decreased to 40 s and a removal efficiency of 81.0% was observed for the xylene concentration of 0.26 g m^−3^ (IL = 23.3 g m^−3^ h^−1^). With the sudden increase in the loading rate (84.9 g m^−3^ h^−1^), the removal efficiency of xylene decreased to 53.6%, but it recovered to 68.2% after 3 days. Similar to the previous phases, the biofilter experienced high xylene loading rates ranged from 192.7 to 204.6 g m^−3^ h^−1^ during days 90–95 and in accordance with this operating condition, an average xylene removal efficiency of 30.3 ± 2.2% was obtained. At the end of this phase, the xylene concentration was lowered and the removal efficiency recovered to 89% for loading rate of 22.2 g m^−3^ h^−1^.

In this study, a removal efficiency of 98% was obtained under steady state condition for the inlet concentration of 1.34 g m^−3^ at EBRT of 90s. Comparatively, Saravanan and Rajamohan [1] observed a maximum xylene removal efficiency of 50% for the inlet concentration of 1.2 g m^−3^ at an EBRT of 88.2 s in a biofilter packed with press mud.

The results obtained during this study indicate that the xylene removal efficiency decreased either by an increase in xylene concentration in the entering air or by a decrease in EBRT. At lower EBRTs, the microbial population on the surface of media gets less contact time for the degradation of xylene. Besides, the increase in xylene loads towards the highest levels during the last two phases, especially phase II, occurred in a more rapid manner than that of the phase I. Then, there was more time for microorganisms to adapt the increased xylene at phase I.

**Figure 4:**
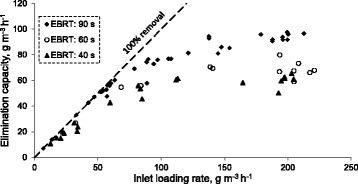
**Effect of xylene inlet load on the elimination capacity of biofilter.**

The elimination capacity (EC), which is the main parameter to describe the biofilter performance, is plotted as a function of the inlet load in Figure 4. Two different regimes are identified, as previously described in the literature [21,22]; the diffusion limitation regime (DLR) and the reaction limitation regime (RLR).

In case of EBRT 90 s; (i) under DLR, the elimination capacity increased to 94.3 g m^−3^ h^−1^ when the inlet loading rate was increased to 137.8 g m^−3^ h^−1^. In DLR, an increase in the inlet concentration of xylene enhances the transfer rate of this pollutant from the gas-phase to the biofilm and more microorganisms actively participate in the biodegradation process. (ii) under RLR, the EC stabilized around 93.9 gm^−3^ h^−1^ for inlet loadings beyond 178.8 g m^−3^ h^−1^ and up to 212.8 gm^−3^ h^−1^. Presumably, the RLR occurs when the amount of active xylene-specific microorganisms is insufficient to degrade all the gas-phase xylene that could possibly be transferred to the biofilm. Under this circumstance, the biofilm would be fully saturated with xylene and the metabolic activity of the cells in the biofilm would be the rate limiting step [3]. The maximum elimination capacity of the biofilter was 97.5 g m^−3^ h^−1^ for inlet xylene load of 199.5 g m^−3^ h^−1^ at EBRT of 90 s. The biofilter performance at EBRTs of 60 s and 40 s had similar trends to that of the EBRT of 90 s, with lower EC values. The maximum ECs of 79.8 and 65.5 g m^−3^ h^−1^ were achieved at EBRTs of 60 and 40 s, respectively. Jorio et al. [23] obtained maximum xylene elimination capacities of 67, 52, and 41 g m^−3^ h^−1^ at EBRTs of 158.9, 90.8, and 63.6 s, respectively, in a conventional biofilter packed with peat mixed with structuring and conditioning agents and initially inoculated with a microbial consortium. In other hand, Gallastegui et al. [24] reached a p-xylene elimination capacity as high as 130 g m^−3^ h^−1^ at high EBRTs ranging from 180 to 270 s in a biofilter packed with pelletised sawdust and pig manure.

Higher elimination capacities at EBRT of 90 s compared to EBRTs of 60 and 40 s (for the same loading rate) may be attributed to the higher residence time of carrier gas, and availability of higher concentration of xylene at higher EBRTs in a given inlet loading rate.

### Xylene profile along the biofilter length

**Figure 5:**
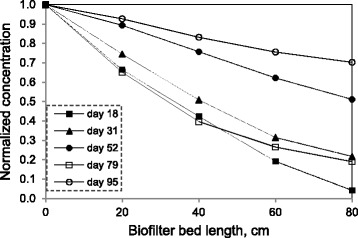
**Normalized xylene concentration profiles along the bed length of biofilter at various days of operation.**

The typical profiles of xylene along the length of the biofilter for different inlet concentrations at EBRTs of 90 and 40 s are illustrated in Figure 5. At day 18 (EBRT 90s), nearly 34% of the inlet xylene was removed in the first 0.2 m of the biofilter, 58% at 0.4 m, 81% at 0.6 m and 96% at 0.8 m of the biofilter height for an inlet xylene concentration of 1.34 g m^−3^ (loading rate of 53.8 g m^−3^ h^−1^). A somewhat similar profile was observed for day 79 (EBRT 40s), where xylene concentration and loading rate were 0.26 g m^−3^ and 23.3 g m^−3^ h^−1^, respectively. Therefore, in moderate inlet xylene concentrations or loading rates, compared to those reported in the literature using biofilter for handling xylene polluted air [1,3], removal is more efficient in the lower parts of the biofilter than in the upper parts. This could be explained by the presence of more carbon source (xylene) in sections near the inlet of the filter bed, which causes a higher metabolic reaction [25]. Since the biofilter was operated in the up-flow mode, then the highest local concentration of xylene was at the bottom of the bed and the lowest local concentration of xylene was at the top of the bed. With an increase in inlet xylene concentration to 5.0 g m^−3^ at day 52 (EBRT 90s), all sections had almost similar removal efficiencies ranging from 10.7% to 13.6%. Likewise, all sections showed somewhat similar performance with an average removal efficiency of 7.4% ± 1.7% at day 95 (C_in_ = 2.27 g m^−3^, EBRT 40s). The xylene loads at days 52 and 95 were 199.5 and 204.6 g m^−3^ h^−1^, respectively, which lay in reaction limitation regime (Figure 4). The xylene removal rate under this regime is nearly independent from inlet concentration variations.

At day 95, the inlet xylene concentration was about that of the day 31 (C_in_ = 2.43 g m^−3^, IL = 97.1 g m^−3^ h^−1^), while xylene load was about that of the day 52. As shown in Figure 5, the xylene removal profile at day 95 was very similar to that of the day 52. This indicates that the xylene removal profile along biofilter length is more dependent on the inlet load than on the inlet concentration.

### Carbon dioxide production

In biofiltration process, the CO_2_ production is an important parameter indicating the degree of VOCs degradation, because VOCs are aerobically biodegraded into water and carbon dioxide and utilized as carbon source for the microbial growth [16]. For complete chemical oxidation of xylene to water and CO_2_, the mass-ratio of CO_2_ produced to xylene degraded should be 3.32, according to the following stoichiometric reaction:

5 $$ {C}_8{H}_{10}+10.5\ {O}_2\to\ 8C{O}_2 + 5{H}_2O $$

**Figure 6:**
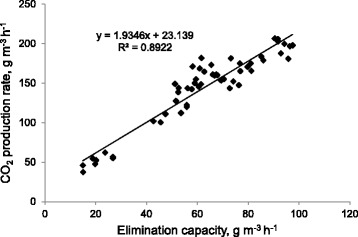
**Relationship between the carbon dioxide production rate (PCO**_**2**_ **) and xylene elimination capacity (EC).**

The carbon dioxide production rate (PCO_2_) as a function of the xylene elimination capacity (EC) is shown in Figure 6. The alignment of the data series indicates that the quantity of carbon dioxide produced strongly correlated with the quantity of xylene eliminated and a linear regression provides the following equation:

6 $$ PC{O}_2=1.93EC+23 $$

Accordingly, the value of the slope in equation [6] shows that about 60% of the eliminated xylene was converted to CO_2_. Wu et al. [26] obtained a mass ratio of PCO_2_/EC of 1.65 when they treated p-xylene in a hybrid biofilter with an added nutrient solution containing ammonium salts. Li et al. [27] reported that 62% of removed xylene in a bacterial and fungal biofilter was converted to CO_2_.

The discrepancy observed in CO_2_ production in comparison with the case of complete chemical oxidation of xylene can be mainly explained by the biomass production. In addition, some of the CO_2_ produced may partly accumulate in the liquid-phase in the form of $\mathrm{H}\mathrm{C}{\mathrm{O}}_3^{\hbox{-} },{\mathrm{H}}_2\mathrm{C}{\mathrm{O}}_3,\;\mathrm{and}\;\mathrm{C}{\mathrm{O}}_2^{\left(\hbox{-} 3\right)}$ [26].

By taking into account a general biomass composition formula as C_5_H_7_NO_2_ and PCO_2_/EC ratio of 1.93 (equation 6) (ignoring the CO_2_ accumulated in the leachate), and considering ammonium as nitrogen source, equation [5] could be re-written as:

7 $$ {C}_8{H}_{10}+7.32{O}_2+0.67N{H}_4\to 0.67{C}_5{H}_7{O}_2N+4.65C{O}_2+3.99{H}_2O $$

Therefore, 0.71 g of dry biomass was produced per g of xylene consumed, which corresponds to a biomass yield coefficient value of 0.42 gC dry mass synthesized per gC xylene degraded.

### Microbial aspects

**Figure 7:**
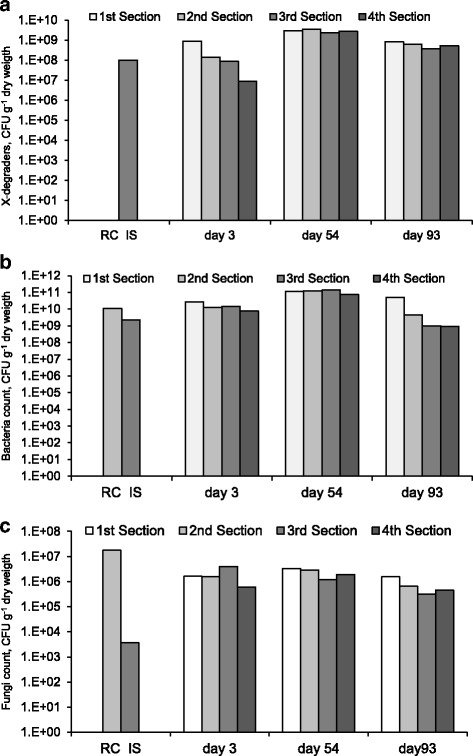
**The microbial counts of (a) xylene degraders, (b) total bacteria, and (c) total fungi in the biofilter bed at different days of operation (RW: Raw Compost, IS: Inoculated Scoria).**

Microbial counts in the filter material were regularly achieved for the follow-up of the microbial growth intensity inside the biofilter. The xylene-degraders, bacteria, and fungi counts in the packing material before biofilter assembly and in the samples withdrawn from each section of the filter bed at different days of biofilter operation are presented in Figure 7(a), (b), and (c). Inoculated scoria contained 10^8^ CFUs of xylene degraders per gram dry weight, while no CFU was detected in one gram dry raw compost and scoria. This is taken as an evidence of the appropriate development of the inoculum, which probably resulted in good biofilter performance during the initial days of operation.

At day 3, the counts of xylene-degraders decreased around two orders of magnitude from bottom to the top of biofilter, which can be related to the complete removal of xylene at the first two sections. The xylene degraders increased by 10-fold to an average count of 2.87 × 10^9^ ± 4.8 × 10^8^ CFU g^−1^ at day 54 of operation and then decreased by 5-fold to 5.82 × 10^8^ CFU g^−1^ at day 93. These values are higher than those reported for p-xylene degraders in the biofilters packed with food waste compost (1.28 × 10^8^ g^−1^ of dry compost) and pig manure compost (2.58 × 10^7^ g^−1^ of dry compost) [20]. The microbial counts for xylene degraders follow xylene elimination capacities, which were 33.0, 93.7, and 50.2 g m^−3^ h^−1^ at days 3, 54, and 93, respectively. In addition, the number of xylene degraders in all four sections of the biofilter were within the range of the same order of magnitude at days 53 (9 log CFU g^−1^) and 93 (8 log CFU g^−1^), which are related to approximately similar performance of the sections at high inlet xylene loads.

The comparison of the number of microbial cells (average ratio of bacterial to fungal CFUs =2.4 × 10^4^:1) implies that the bacteria were the dominant microorganisms responsible for the degradation of xylene in the biofilter. Irrespective of the day of sampling and biofilter section, the average total bacterial count was 4.8 × 10^10^ CFU (gram of dry mass)^−1^ containing 10.5% xylene degraders.

**Figure 8:**
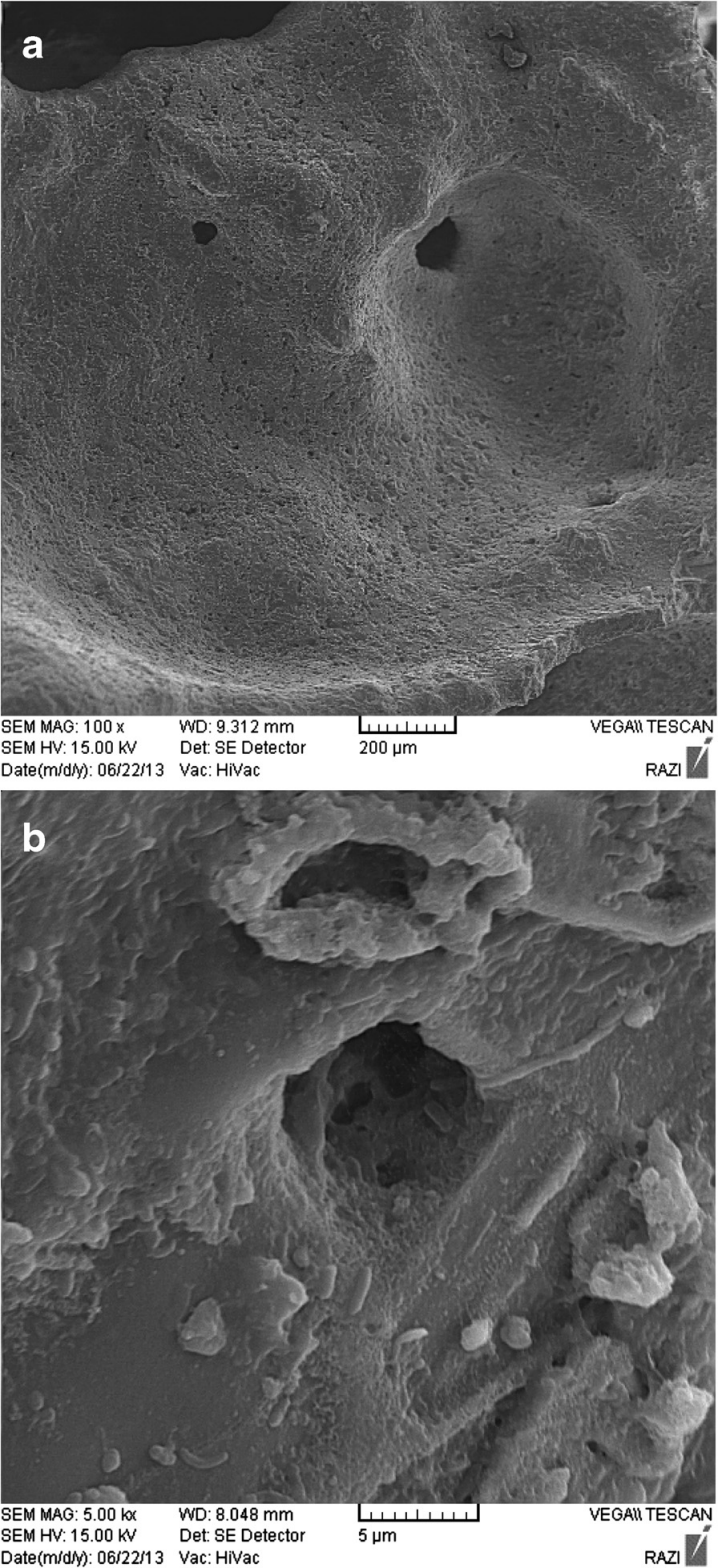
**SEM micrographs of (a) raw scoria (×100) and (b) filter bed taken from 3** ^**rd**^ **section at the end of biofilter operation (×5000).**

A low magnification SEM image revealed that the scoria is very porous, and it showed a raw surface with big pores, which allowed the microbial attachment (Figure 8(a)). In addition, a sample was taken from the third section of biofilter at the end of operating period and analyzed using SEM. A dense biofilm covered the surface of the scoria and extended to the pores (Figure 8(b)). While the morphology is difficult to be observed, some coccoid and rod-shaped bacteria embedded in a matrix of extracellular polymeric substances (EPS) are present. Although such dense biofilm limits the diffusion of pollutants toward the inner particles, it may improve the resistance of microorganisms against high concentrations and shock loads of xylene.

### Temperature and pressure drop

**Figure 9:**
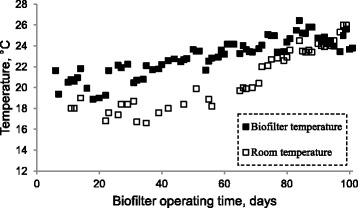
**The biofilter bed and room temperature variations over biofilter operating time.**

The average bed temperature changed from 18.9 to 26.4°C through the experiment, which is within the normal range reported for the biofiltration of gas phase VOCs [28]. The VOCs biodegradation by microorganisms in biofilters is an exothermic process, which increases the temperature inside of the biofilters [29]. Although the temperature of inlet gas sometimes increased up to 5.6°C along biofilter, no significant correlation was found between EC and temperature (p = 0.675). This could be due to the heating of the inlet gas stream in cool conditions and large changes in ambient air temperatures, ranging from 16.6 to 26.0°C. The average filter bed temperature was mainly subject to room temperature (Figure 9) as well as a significant correlation was found between them (p < 0.001, R^2^ = 0.735).

The pressure drop values were less than 4 mm H_2_O during the entire experiments. In addition, negligible compaction and deterioration of the bed was observed, indicating a good mechanical strength of the media. The low pressure drop, which is the prime prerequisite for biofilter packing material, could be related to low velocity of carrier gas, the filter media characteristics, irrigation strategy and the nature of microbial community within biofilter. By volume, about 75% of the filter material was scoria (6–13 mm particle size), which is an inert media. One major advantage of inert material on organic ones is the low pressure drop due to minimum compaction over time and good air distribution. Excess biomass accumulation is a major factor causing an increase in the pressure drop [30]. Nevertheless, adequate irrigation of the filter bed made the removal of the excess of biomass possible [23]. Here the filter bed was irrigated with 1 liter nutrient solution, where, 728 and 1620 mg TOC were discharged from the system at days 59 and 94, respectively. The filter bed was dominated by bacteria. Usually pressure drop in bacterial biofilters is lower than that of observed in fungal biofilters. This is because of the occupation of the free space by the mycelia. Estrada et al. [31] reported that the bacterial biofiltration treating a VOC mixture exhibited a final pressure drop of 60% lower than that of the fungal biofilter due to mycelial growth.

## Conclusions

A biofilter packed with scoria/compost was employed for the removal of xylene vapors. The best performance of biofilter (EC_max_ = 97.5 g m^−3^ h^−1^) was observed at the highest EBRT, that may be due to higher residence time of carrier gas. The CO_2_ production rate and the distribution of microbial populations in the biofilter were well correlated with the xylene removal rates, indicating the biodegradation of xylene in the biofilter. The packing media provided low pressure drop and long term stability, demonstrating its potential as media in the biofiltration of VOCs.
